# The EASI model: A first integrative computational approximation to the natural history of COPD

**DOI:** 10.1371/journal.pone.0185502

**Published:** 2017-10-10

**Authors:** Alvar Agustí, Albert Compte, Rosa Faner, Judith Garcia-Aymerich, Guillaume Noell, Borja G. Cosio, Robert Rodriguez-Roisin, Bartolomé Celli, Josep Maria Anto

**Affiliations:** 1 Respiratory Institute, Hospital Clinic, Universitat de Barcelona, Barcelona, Spain; 2 Institut d’Investigacions Biomediques August Pi i Sunyer (IDIBAPS), Barcelona, Spain; 3 CIBER Enfermedades Respiratorias (CIBERES), Barcelona, Spain; 4 ISGlobal, Centre for Research in Environmental Epidemiology (CREAL), Barcelona, Spain; 5 IMIM (Hospital del Mar Medical Research Institute), Barcelona, Spain; 6 Universitat Pompeu Fabra (UPF), Barcelona, Spain; 7 CIBER Epidemiología y Salud Pública (CIBERESP), Barcelona, Spain; 8 Hospital Universitari Son Espases-IdISBa, Palma de Mallorca, Spain; 9 Harvard Medical School, Boston, Massachussets, United States of America; National and Kapodistrian University of Athens, GREECE

## Abstract

The natural history of chronic obstructive pulmonary disease (COPD) is still not well understood. Traditionally believed to be a self-inflicted disease by smoking, now we know that not all smokers develop COPD, that other inhaled pollutants different from cigarette smoke can also cause it, and that abnormal lung development can also lead to COPD in adulthood. Likewise, the inflammatory response that characterizes COPD varies significantly between patients, and not all of them perceive symptoms (mostly breathlessness) similarly. To investigate the variability and determinants of different “individual natural histories” of COPD, we developed a theoretical, multi-stage, computational model of COPD (EASI) that integrates dynamically and represents graphically the relationships between exposure (*E*) to inhaled particles and gases (smoking), the biological activity (inflammatory response) of the disease (*A*), the severity (*S*) of airflow limitation (FEV_1_) and the impact (*I*) of the disease (breathlessness) in different clinical scenarios. EASI shows that the relationships between *E*, *A*, *S* and *I* vary markedly within individuals (through life) and between individuals (at the same age). It also helps to delineate some potentially relevant, but often overlooked concepts, such as disease progression, susceptibility to COPD and issues related to symptom perception.

In conclusion, EASI is an initial conceptual model to interpret the longitudinal and cross-sectional relationships between *E*, *A*, *S* and *I* in different clinical scenarios. Currently, it does not have any direct clinical application, thus it requires experimental validation and further mathematical development. However, it has the potential to open novel research and teaching alternatives.

## Introduction

The natural history of chronic obstructive pulmonary disease (COPD) is still not well understood. Traditionally believed to be a self-inflicted disease by smoking [1], now it is well established that not all smokers develop the disease [2], that other inhaled pollutants different from cigarette smoke can also cause COPD [3], and that early-life events can jeopardize lung development and lead to COPD in adulthood [4–6]. Likewise, albeit inflammation is considered a key pathogenic player [7], the type and severity of the inflammatory response (the biological activity of the disease) varies significantly between patients [8, 9]. Finally, the clinical impact of the disease (how the patient perceives the symptoms originated by the disease, mostly breathlessness) also varies across patients with similar lung function impairment [10, 11].

Computational models can help to understand complex biological problems by offering a theoretical framework where to explore the relationships amongst different variables [12]. They have, therefore, the potential to generate novel hypotheses that can be later tested experimentally [12, 13]. Here, we hypothesize that the natural history of COPD is the end-result of a complex multi-stage process (environmental exposures, biological response, lung structure and function deterioration and symptom perception), and that each of these stages exhibits large individual variability that result in different natural history trajectories. As a first attempt to explore this hypothesis, we developed an individualized, multi-stage computational model of COPD (named EASI) that explores, integrates and displays graphically the dynamic relationships in a given individual between *E*xposure (smoking), *A*ctivity (Inflammation), *S*everity (as assessed by the expired volume of gas in the first second of a forced spirometry maneuver—FEV_1_) and *I*mpact of the disease (dyspnea). We explicitly acknowledge that, at this stage of development, EASI cannot be used to predict the course of the disease in a given individual, nor the response to any therapeutic intervention. By contrast, EASI is envisaged as a theoretical, conceptual computational model that begins to explore the relationships between *E*, *A*, *S* and *I* in different clinical scenarios to facilitate the design of appropriate field studies that can confirm or dispute the predictions of the model [13].

## Methods

**Fig 1:**
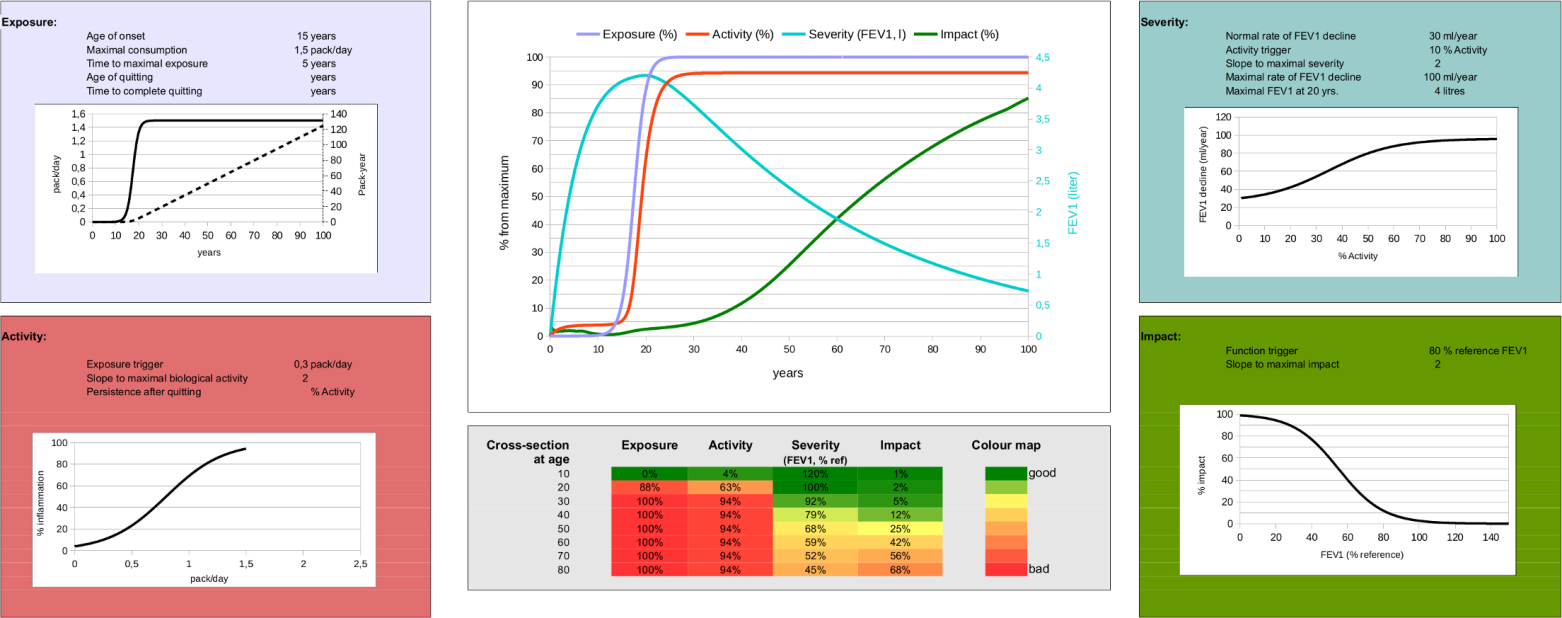
Graphic display of the EASI model structured around four modules (*E*xposure (top left), *A*ctivity (bottom left), *S*everity (top right), *I*mpact (bottom right)), each of which presents the parameter values used to calculate it, and the relevant steady-state activation functions linking inputs from previous module (X axis) to module outputs (Y axis). In *E*, the black solid line indicates daily smoking exposure (pack/day; left Y axis) as a function of age (X axis), whereas the dashed line corresponds to the cumulative smoking exposure (pack-years, right Y axis) of that particular individual. The EASI model also includes two central panels. The top one presents a longitudinal summary of the age-related trajectories of Exposure (blue line), Activity (red line), FEV1 (cyan line; right Y panel) and Symptoms (green line). The bottom (centre) panel presents a heat-map of these same four variables by decade. This particular example illustrates the EASI relationships for a susceptible continuous smoker (Table 1 details the parameter values used here). For further explanations, see text.

As shown in Fig 1, EASI has 4 stage modules (*E*xposure, *A*ctivity, *S*everity and *I*mpact), each of them defined by 4 dynamic variables (*E*(*t*), *A*(*t*), *S*(*t*), *I*(*t*)) and 15 input parameters (Table 1) which were based on published data when available [6, 14, 15] or, in its absence, clinical experience. The mathematical assumptions and specific differential equations used in each module are detailed in the S2 File. In brief, *E(t)* models smoking related parameters as the product of two sigmoid (logistic) functions of time (for the initiation and quitting of smoking, respectively), whereas *A(t)*, *S(t)* and *I(t)* are modeled using first-order linear ordinary differential equations that depend on one another in a hierarchical, feed forward way (*A* depends on *E*, *S* on *A*, and *I* on *S*). These differential equations were integrated using an Euler method with time step Δ*t* = 0.2 years. The model was written as an interactive spread sheet using the freely-available package LibreOffice (http://www.libreoffice.org). This fully workable spread sheet is available in the S1 File, licensed under a Creative Commons Attribution 4.0 International License. EASI parameters were calibrated *in silico* using a Matlab custom code (The Mathworks, Inc.) by comparison with available FEV1 population data, both for smoking and non-smoking populations, as detailed in the S2 File.

**Table 1:** Input parameters (n = 15) used to generate the six different clinical scenarios analyzed. Bold italic figures highlight the changes introduced in the EASI model with respect to the previous scenario (i.e., left column).

	(1) Susceptible continuous smoker	(2) Susceptible quitter at 45 years	(3) Susceptible quitter at 65 years	(4)Persistent inflammation despite quitting	(5) Abnormal lung development	(6) Poor perceiver
1. Age of smoking onset, yrs.	15	15	15	15	15	15
2. Maximal exposure (packs/day)	1.5	1.5	1.5	1.5	1.5	1.5
3. Time to max. exposure, yrs.	5	5	5	5	5	5
4. Age of quitting, yrs.	Non-quitter	***45***	***65***	***45***	***Non-quitter***	Non-quitter
5. Time to complete quitting, yrs.	**-**	***1***	1	1	**-**	-
6. Activity trigger, pack/day	0.3	0.3	0.3	0.3	0.3	0.3
7. Slope to maximal activity	2	2	2	2	2	2
8. Persistence after quitting, % activity	Non-quitter	***0***	0	***40***	***Non-quitter***	Non-quitter
9. Normal rate of FEV_1_ decline, ml/yrs.	30	30	30	30	30	30
10. Activity trigger, % activity	10	10	10	10	10	10
11. Slope to maximal severity	2	2	2	2	2	2
12. Maximal rate of FEV1 decline, ml/yrs.	100	100	100	100	100	100
13. FEV_1_ at 20 yrs. of age, liters	4 (100% ref.)	4 (100% ref.)	4 (100% ref.)	4 (100% ref.)	***3*.*0 (75% ref)***	***4 (100% ref*.*)***
14. Impact trigger, % reference FEV_1_	80	80	80	80	80	***50***
15. Slope to max. Impact	2	2	2	2	2	2

## Results

### Longitudinal, age-related, multi-stage dynamics in different clinical scenarios

Fig 1 presents the graphic EASI representation of a theoretical (but frequent) case of a susceptible, continuous long-life smoker who develops COPD in adult life (Table 1 presents the 15 specific input parameters used to compute this particular scenario). Of note, this theoretical individual: *(1)* starts to smoke at the age of 15 yrs., achieves a maximal exposure of 1.5 pack-yr. within 5 yrs., and never quit or modify the daily dose of smoking; (Fig 1, top left panel); *(2)* has normal lung development, as shown by an FEV_1_ at the age of 20 years of 100% ref. [16] (Fig 1, heat map in bottom central panel); and, *(3)* begins to perceive dyspnea from a value of FEV_1_ <80% ref (Fig 1, bottom right panel). The results of the relationships between the *E*, *A*, *S* and *I* modules are displayed in two age-related panels (Fig 1, central panels) that summarize the “natural history” of COPD in this particular individual. The upper central panel shows that the onset and persistence of smoking (blue line) induced a rapid and sustained inflammatory activity (red line) that is associated with a progressive loss of FEV_1_ (cyan line, right Y axis), starting at the age of 30 years approximately, and reaching a FEV_1_ 45% of reference at the age of 80 (see heat-map in bottom central panel), with a progressive perception of symptoms (green line) from the fifth or sixth decade of age (reaching 68% of maximal impact *I* at 80 years of age).

**Fig 2:**
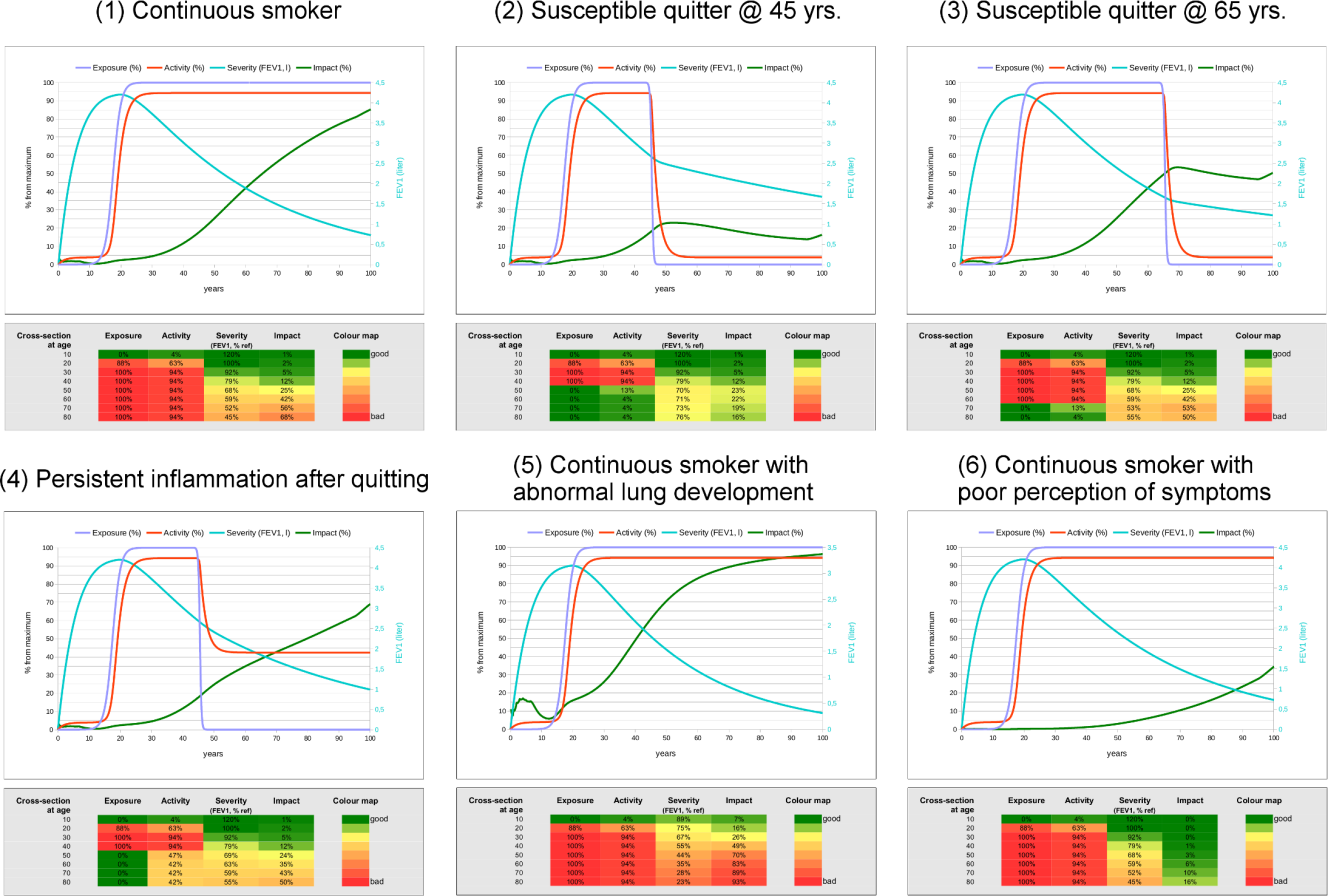
Comparison of six different, potentially relevant clinical scenarios. *Panel 1*, Susceptible continuous smoker (same as Fig 1); *Panel 2*, Susceptible quitter at 45 years of age; *Panel 3*, Susceptible quitter at 65 years of age; *Panel 4*, Persistent inflammation after quitting; *Panel 5*, Abnormal lung development; and, *Panel 6*, Poor perceiver. In each of these five different scenarios, top panels present a longitudinal summary of age-related trajectories of Exposure (blue line), Activity (red line), Severity (FEV1, cyan line) and perceived impact of the disease by the patient (green line), whereas bottom panels show the corresponding heat-maps by decade. Table 1 details the specific parameter values to each module used to generate each of these five scenarios, and Fig 1 and S4–S7 Figs displayed each of them graphically in detail. For further explanations, see text.

Fig 2 compares this theoretical scenario (*Panel 1*) with 5 other potential “natural histories” (*Panels 2–6*). Table 1 details the specific parameter values used to simulate each of them. *Panel 2* assumes that the same individual shown in *Panel 1* now quits smoking at the age of 45 yrs. (see S4 Fig for details). Note that, now, the blue (*E*) and red (*A*) lines fall to zero as a consequence of smoking cessation and inflammation remission, whereas the *S* (cyan line; FEV_1_) and *I* (green line) slopes become less steep. *Panel 3*, presents a smoker quitting smoking later in life (at 65 yrs. (see S5 Fig for details) and shows that, in keeping with previously published data [2], improvements of *S* and *I* are less marked after quitting at older age (compare *Panels 2* and *3*). *Panel 4* is identical to *Panel 2* (see Table 1 and S6 Fig for details), except that, as suggested in the literature [17], EASI now assumes that despite quitting smoking, inflammatory activation persists at 40% of the maximal value (note that the red line now plateaus, and *S* and *I* continue to deteriorate). *Panel 5* (see S7 Fig for details) is similar to Panel 1, except that now the model assumes that lung development was impaired in early life, so maximal lung function at 20 years of age was 75% of maximal normal function (see % FEV_1_ values in the corresponding heat map). Finally, *Panel 6* (see S8 Fig for details) is also similar to *Panel 1*, but now the patient perceives dyspnea poorly, as illustrated by the comparison of the green line *(I)* in Panels 6 *vs*.1.

### Cross-sectional patterns of trajectories

In clinical practice, patients generally consult when they perceive symptoms in late adulthood. At that time, life-long *longitudinal* information is lacking almost invariably, so practicing physicians need to integrate available *cross-sectional* information (basically *E*, *S* and *I*, since *A* is rarely, if ever, measured). It was therefore of interest to compare the *cross-sectional pattern* (by decade of age) of the six different scenarios described in Fig 2. Their visual comparison (see heat-maps in Fig 2) illustrates that the *pattern* of *E*, *A*, *S* and *I* relationships varies greatly *within* each clinical scenario at different ages (e.g. 30 *vs*. 60 yrs.), as well as *between* clinical scenarios at the same age (e.g., 60 years, when most COPD patients are diagnosed in the clinic).

**Fig 3:**
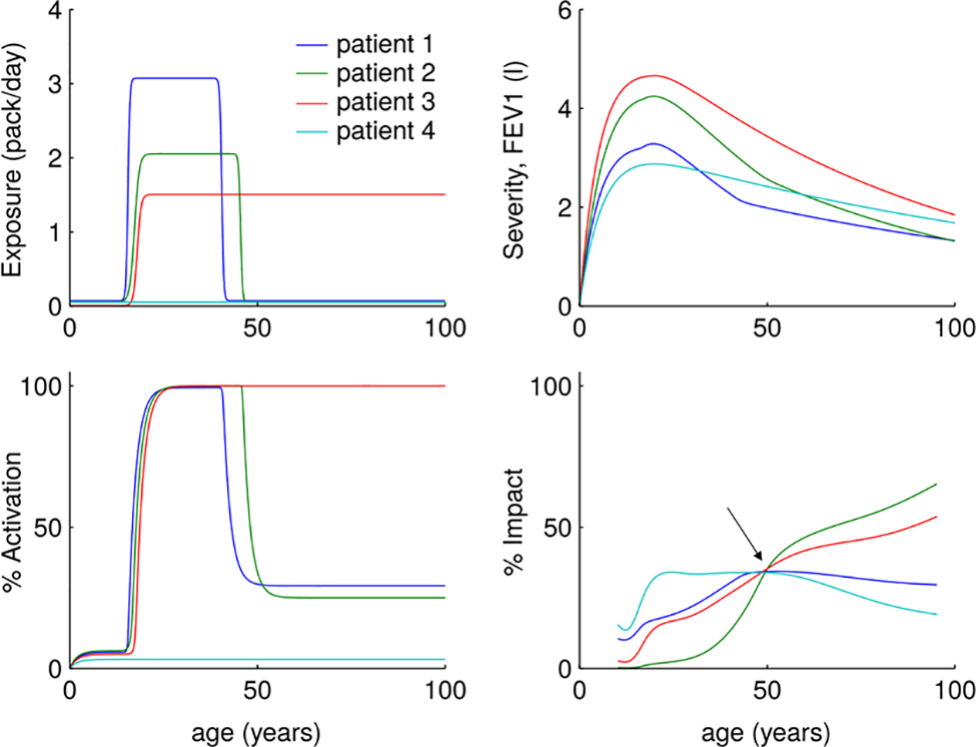
Age-related Exposure, Activity, Severity and Impact trajectories of four hypothetical patients. Arrow (bottom right panel) highlights that all four of them had the same *I* at 50 years of age (when they consult with the physician). Yet, their *E*, *A*, and *S* trajectories are quite different. Note that one patient (#4) is a never-smoker individual with low lung function at early age, who may be diagnosed of COPD later in life (60 years). For further explanations, see text.

Finally, we modeled (see Table 2 for parameter values) four different hypothetical patients consulting at the age of 50 years with a similar level of symptoms (Fig 3, arrow) but remarkably different individual life-time *E*, *A*, *S* and *I* trajectories. Fig 3 includes a non-smoker individual (Patient 4) with low lung function at early age (Table 2) who, according to *S* only would be likely to be diagnosed of COPD later in life [6].

**Table 2:** Parameter values used to generate the EASI relationships presented in the four different hypothetical patients shown in Fig 3.

	Patient 1	Patient 2	Patient 3	Patient 4
**Exposure Module**				
1. Age of smoking onset, yrs.	15	16	17	Non smoker
2. Maximal exposure (packs/day)	3	2	1.5	Non smoker
3. Time to max. exposure, yrs.	1	2.5	2	Non smoker
4. Age of quitting, yrs.	40	45	Non-quitter	Non smoker
5. Time to complete quitting, yrs.	1	1	Non-quitter	Non smoker
**Activity Module**				
6. Activity trigger, pack/day	0.3	0.1	0.1	0.35
7. Slope to maximal activity	2	3.5	3.5	2
8. Persistence after quitting, % Activity	25	20	Non-quitter	Non smoker
**Severity Module**				
9. Normal rate of FEV_1_ decline, ml/yrs.	12	35	20	20
10. Activity trigger, % epithelial apoptosis	20	10	30	20
11. Slope to maximal severity	2	2.5	1	1
12. Maximal rate of FEV_1_ decline, ml/yrs.	75	90	75	60
13. FEV_1_ at 20 yrs. of age, liters	3	4	4.5	2.8
**Impact Module**				
14. Impact trigger, % reference FEV_1_	90	85	115	85
15. Slope to max. Impact	1	3	2	2

## Discussion

To our knowledge this is the first computational model to simulate the individual variability of COPD “natural histories” [13]. EASI shows that, in contrast to the current idealized one-size-fits-all nature of COPD paradigms, there can be a large degree of individual variability through time that is often difficult to dissect based on cross-sectional pattern analysis only. Further, it highlights some, potentially relevant but often overlooked, observations related to the pathogenesis of the disease, as discussed below. Yet, it is important to reiterate here that EASI does not pretend to predict the course of the disease in any given patient, nor the response to any therapeutic intervention, in current clinical practice, albeit we recognize that it may eventually have such capacity when appropriately validated and developed.

### Variability of COPD trajectories (i.e., different “natural histories”)

EASI displays the variability and sensitivity to changes of different individual COPD trajectories by showing that the variation of a single parameter (Fig 2) can change them markedly. Needless to say that this is a direct consequence of the way EASI was built, having one module feeding the necessary information to the next one (*feed-forward*). This kind of relationship is likely to occur also in real life, but the situation *in vivo* is much more complex and includes the possibility of *feed-back*, redundancy and a range of potential adaptations [12] that are not considered in the current version of EASI. For example, patients can adapt their daily life activities to minimize symptoms (i.e., *I*) when airflow limitation (i.e., *S*) is severe. Likewise, rehabilitation can have beneficial effects on *I* and, perhaps on *A*, without changing *S*. EASI can be scaled in the future to accommodate all these potential modifiers and others, such as exacerbations of the disease [18]. In this context, it is noteworthy that the single change with the greatest effect on lung function later in life was poor lung function in early adulthood (Fig 2), in line with several recent observations [5, 6, 19].

Likewise, EASI also shows that the relationships between *E*, *A*, *S* and *I* not only vary markedly longitudinally (different scenarios through life) but also cross-sectionally (same scenario across different ages) (Fig 2). In fact, four different hypothetical patients can present in this “virtual clinic” with the same *I* (i.e., symptoms) after having had very different longitudinal trajectories (Fig 3). In essence, this highlights the need of longitudinal studies in well characterized cohorts of patients to understand *in vivo* these different trajectories and, in turn, confirm (or refute) the initial and simple EASI model presented here.

### Disease progression

COPD progression is often assessed by the rate of FEV_1_ decline [1] but recent evidence has demonstrated that only a proportion of patients have enhanced FEV_1_ decline [5, 6]. The EASI model goes further and indicates that (Fig 2) disease progression in COPD can either indicate that the disease continues to be biologically “active” (*A*), with or without continuous exposure (*E*), that it has become more “severe” (*S*) and/or that it has a greater “impact” on the patient (*I*). These different disease components require careful and independent consideration when assessing “disease progression” in COPD.

### Susceptibility to COPD

It is well established that not all smokers develop COPD [1, 2], probably because different genetic backgrounds [20]. Our analysis suggests that this assumption is likely to be much more complex since differences in “susceptibility” may occur for each of the relevant disease stages. For instance, the relationship between *E* (i.e. smoking) and *S* (i.e. FEV_1_) is modulated by *A*, so it is possible that some individuals may be highly susceptible in terms of mounting an inflammatory response that does not translate into clinical disease because their repair mechanisms prevent lung function deterioration. Such individuals would have been traditionally considered a “resistant” smoker despite being highly “susceptible” to the biological effects (*A*) of smoking. This will have to be considered in future studies of COPD “susceptibility”.

### Symptom perception

The clinical impact of any disease, including COPD, depends on how physiologic perturbations are perceived by the individual, and how much these perturbations modify the activities of daily living [21, 22]. In COPD, it has been generally assumed that mild disease (as assessed by the FEV_1_ value) has a minor impact on the patient, whereas the impact is much greater in severe disease [23]. Yet, recent evidence has revealed a more complex picture, since the relationship between the severity of airflow limitation and the level of symptoms reported is poor, and individual variability is enormous [10, 24, 25]. All in all, these observations suggest that there may be poor symptom perceivers among patients with COPD [11], as it is well described in asthma [26, 27]. In fact, EASI predicts that different patients with a similar level of symptoms (*I*) at a given age might have had and will continue to have very different *E*, *A*, *S* and I trajectories (Fig 3).

### Limitations and opportunities

Currently, EASI has several important limitations: *(1)* it is an oversimplification of the natural history of COPD and, for the most part, it lacks experimental validation; *(2)* it includes only four modules and accounts deterministically for their sequential relationships. Each of these modules is susceptible to accommodate other potentially relevant parameters, such as other environmental factors (e.g. occupational exposures or diet), and future EASI iterations could also include other COPD characteristics that can influence the natural history of COPD too, such as the frequency and severity of COPD exacerbations and/or the occurrence of comorbidities; *(3)* the sequential relationships between modules in EASI do not include effect modifiers of module-to-module relationships, such as genetic variants or epigenetic modifications, whose interactions with exposures are likely to influence the individual trajectories of COPD. As a disclaimer, though, the precise genes involved in the pathogenesis of COPD at different time points are unclear, so this first version of EASI considers the genetic background of the individual as a whole. EASI, however, does indeed consider the possibility of modifying lung function achieved at early adulthood [6, 19]; *(4)* EASI modeled *A* pragmatically, without detailing any specific innate and/or acquired mechanism involved [17], because the dynamic interactions of these mechanisms are mostly unknown [28–30]; *(5) S* was modeled on the basis of FEV_1_ only, despite that it is now well established that COPD can associate multiple pulmonary and extra-pulmonary co-morbidities that can contribute to *S* independently [31]; and, *(6)* the sequential relationships between modules could also be influenced by the effects of drugs used to treat COPD, such as those of inhaled bronchodilators (potentially modifying *S*) or corticosteroids (potentially modifying *A*), so future evolutions of the model can include them. No doubt, therefore, EASI requires experimental validation and refinement through an iterative research strategy that combines experimental and modeling data.

Yet, EASI also opens some opportunities: *(1)* it is a first step forward towards the computational modeling (hence better understanding [12, 32, 33]) of the variable “natural histories” that can occur in different COPD patients; and, *(2)* by doing so, it can open novel approaches for research, teaching and, eventually, practice (precision medicine [34]) of COPD.

### Conclusion

EASI is a conceptual, individualized, multi-stage, first-pass, computational model that allows the investigation of the individual relationships between *E*, *A*, *S* and *I* and, as a result, a better understanding of the complexity and heterogeneity of COPD. The current EASI model cannot be directly applied to clinical practice because it requires validation and further refinement, but it points toward a potential path towards COPD precision medicine.

## Supporting information

S1 Fig Distribution of values taken by each of the 15 model parameters in the *in silico* simulation of 1,000 random models in a smoker population [8]. For non-smoker simulations, parameters in the first row were set to 0 for all 1,000 random models. For further explanations, see text.
(TIF)

S2 Fig A heterogeneous population of 1,000 models (parameters as *per* S1 Fig) mimics the mean value (panel A) and variability (panel B) of FEV1 observed experimentally in non-smoker males at different ages [7]. FEV1 decay in persistent smoker model simulations (mean decay across 1,000 models: 50 ml/yr.) is also consistent with experimental data [8]. For further explanations, see text.
(TIF)

S3 Fig Distribution of FEV1 at age 60 years in the random 1,000 models (parameters as shown in S1 Fig) for non-smoker (grey columns) or smoker (white columns) models. Solid vertical line marks the reference FEV1 value reported in the literature for a male of height 1.75 m, whereas dotted vertical line marks its lower limit of normality [7]. For further explanations, see text.
(TIF)

S4 Fig EASI relationships in a susceptible quitter at 45 years of age [12]. For further explanations, see text.
(TIF)

S5 Fig Same as S4 Fig, but the individual now quits smoking at 65 years of age [12]. For further explanations, see text.
(TIF)

S6 Fig EASI relationships in a susceptible quitter (at 45 years of age) in whom inflammation (i.e. disease activity) persists after quitting [4]. For further explanations, see text.
(TIF)

S7 Fig EASI relationships in a continuous smoker who had abnormal lung development early in life [5]. For further explanations, see text.
(TIF)

S8 Fig EASI relationships in a susceptible continuous smoker with poor perception of disease impact [13]. For further explanations, see text.
(TIF)

S1 File Workable spread sheet of the EASI model. (ODS)

S2 File On-line supplement. (DOC)
