# Supplementary figures and images for: The EASI model: A first integrative computational approximation to the natural history of COPD
Source: PLoS One. 2017 Oct 10;12(10):e0185502. doi: 10.1371/journal.pone.0185502 (PMC5634586; doi:10.1371/journal.pone.0185502)

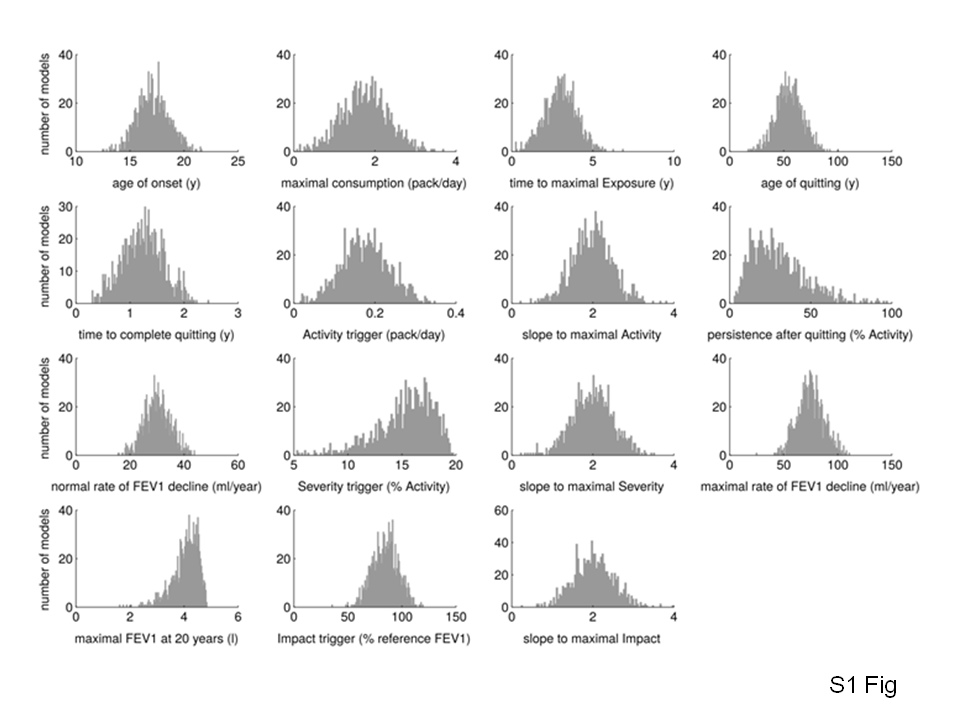

Supplement: S1 Fig — For non-smoker simulations, parameters in the first row were set to 0 for all 1,000 random models. For further explanations, see text. (TIF) [file pone.0185502.s001.tif]

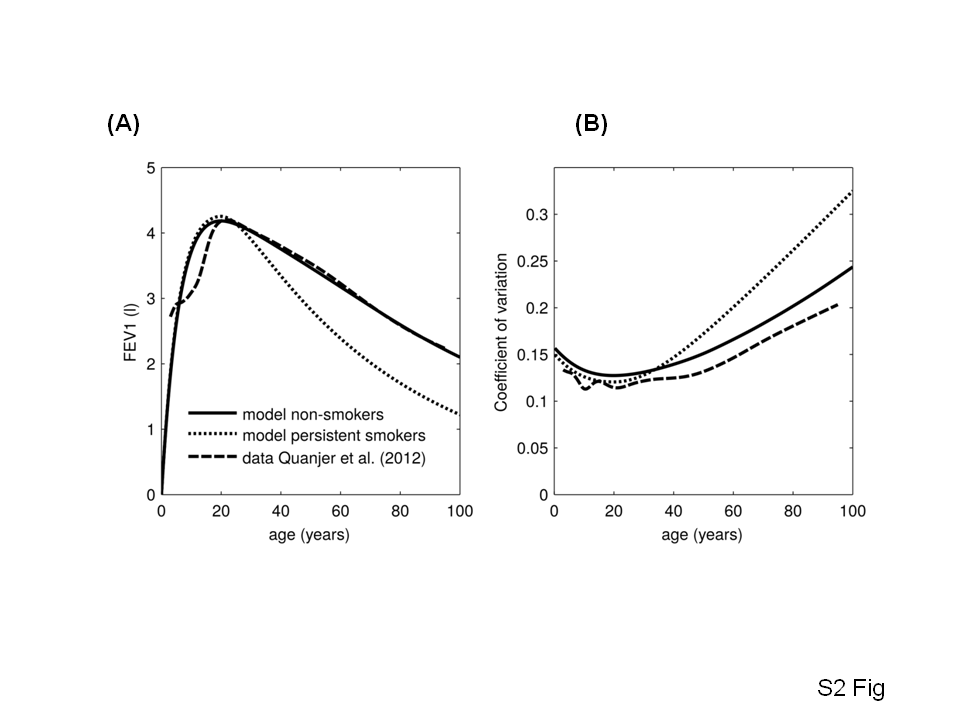

Supplement: S2 Fig — FEV1 decay in persistent smoker model simulations (mean decay across 1,000 models: 50 ml/yr.) is also consistent with experimental data [8]. For further explanations, see text. (TIF) [file pone.0185502.s002.tif]

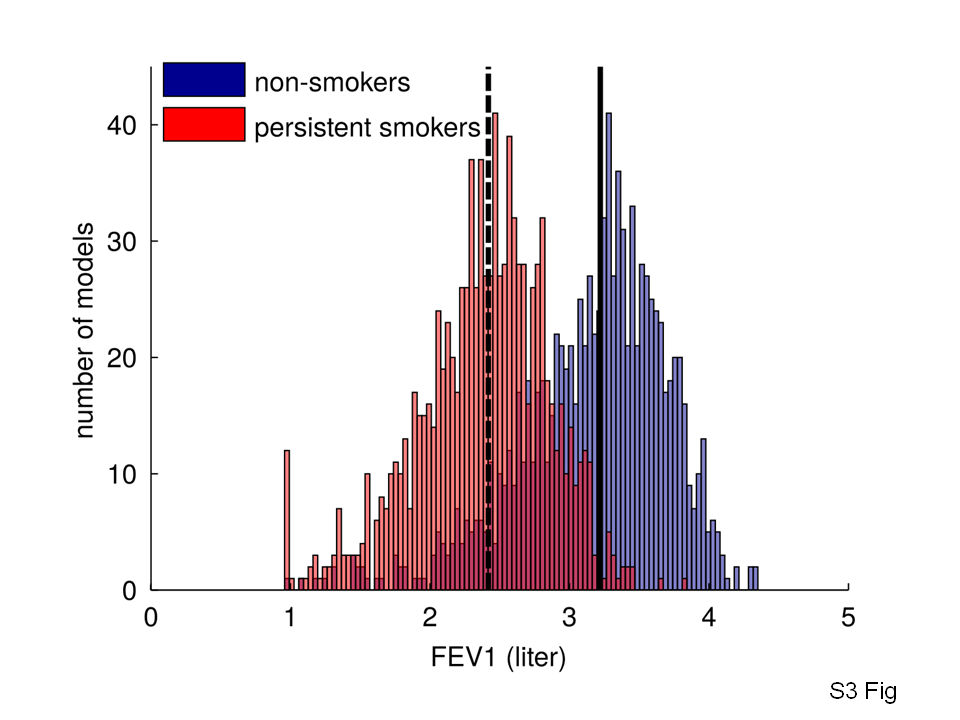

Supplement: S3 Fig — Solid vertical line marks the reference FEV1 value reported in the literature for a male of height 1.75 m, whereas dotted vertical line marks its lower limit of normality [7]. For further explanations, see text. (TIF) [file pone.0185502.s003.tif]

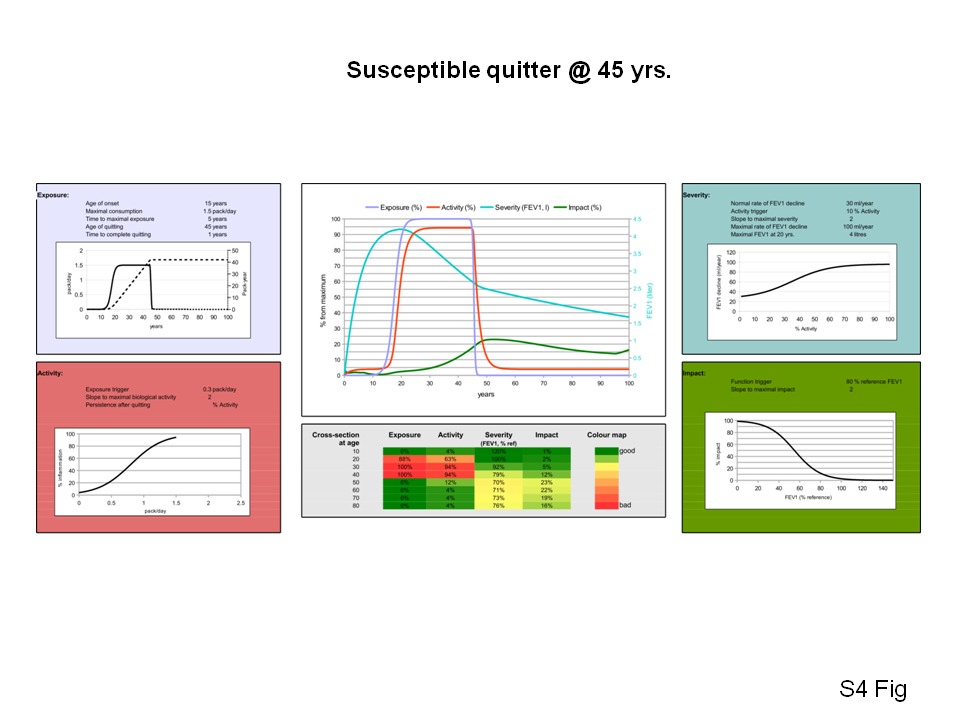

Supplement: S4 Fig — For further explanations, see text. (TIF) [file pone.0185502.s004.tif]

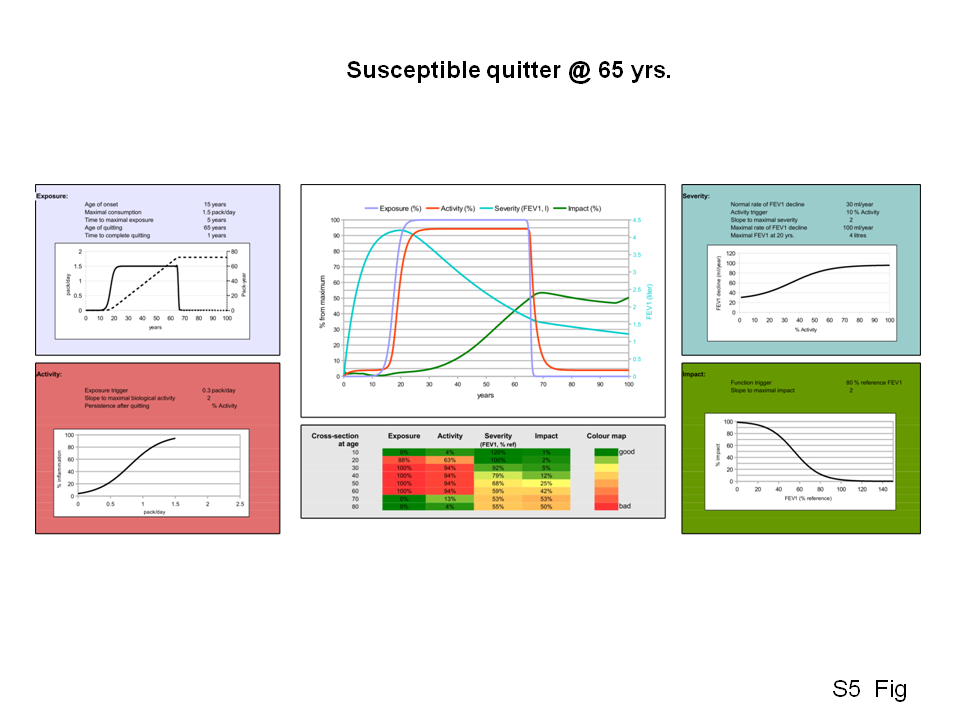

Supplement: S5 Fig — For further explanations, see text. (TIF) [file pone.0185502.s005.tif]

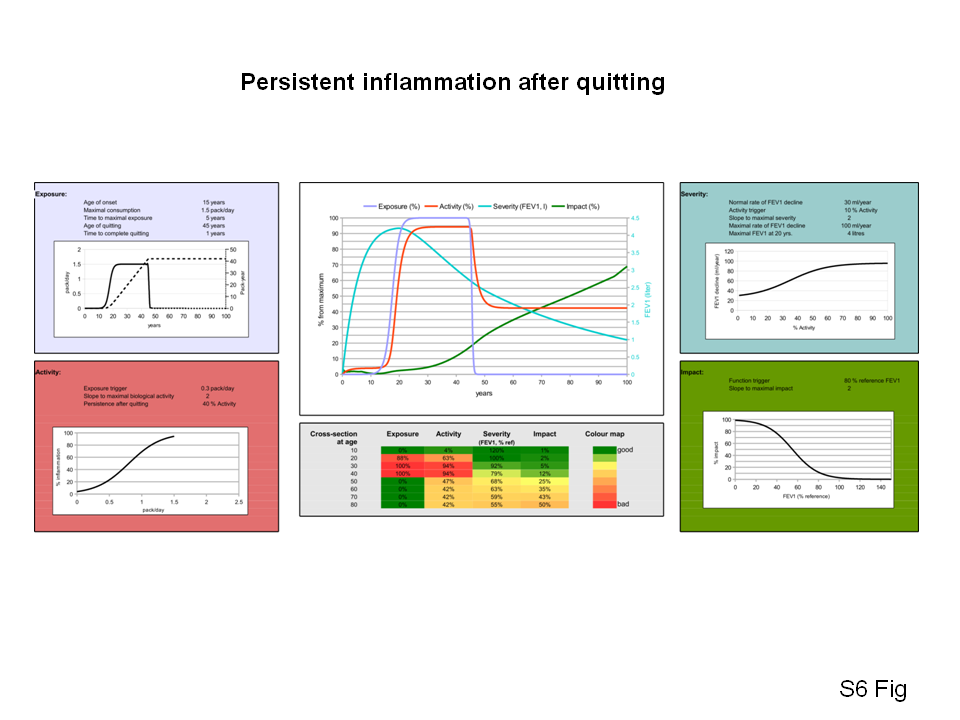

Supplement: S6 Fig — For further explanations, see text. (TIF) [file pone.0185502.s006.tif]

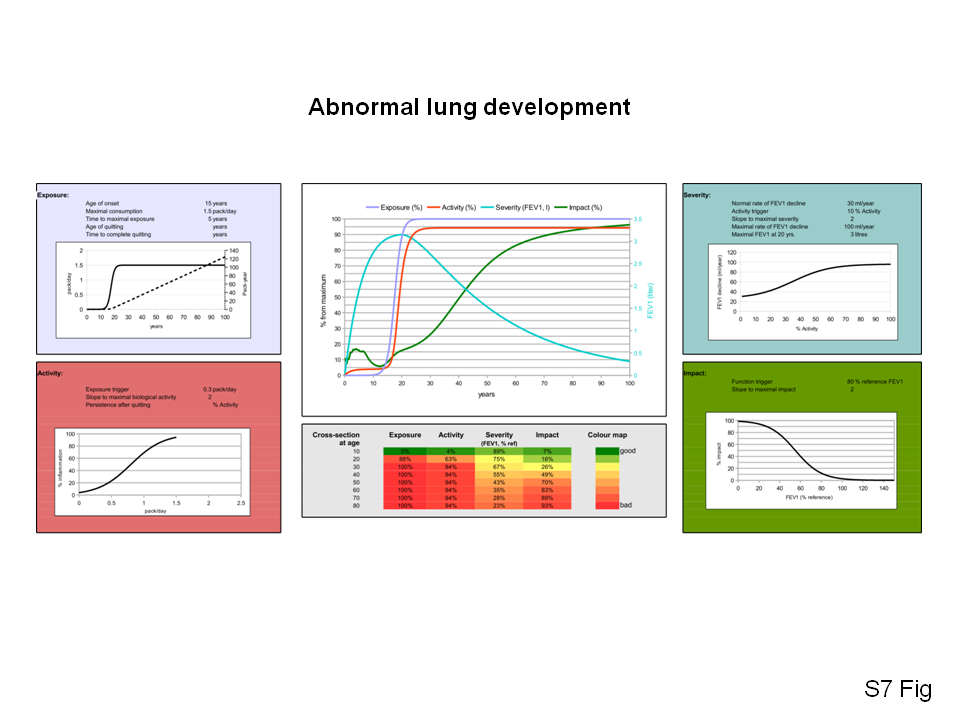

Supplement: S7 Fig — For further explanations, see text. (TIF) [file pone.0185502.s007.tif]

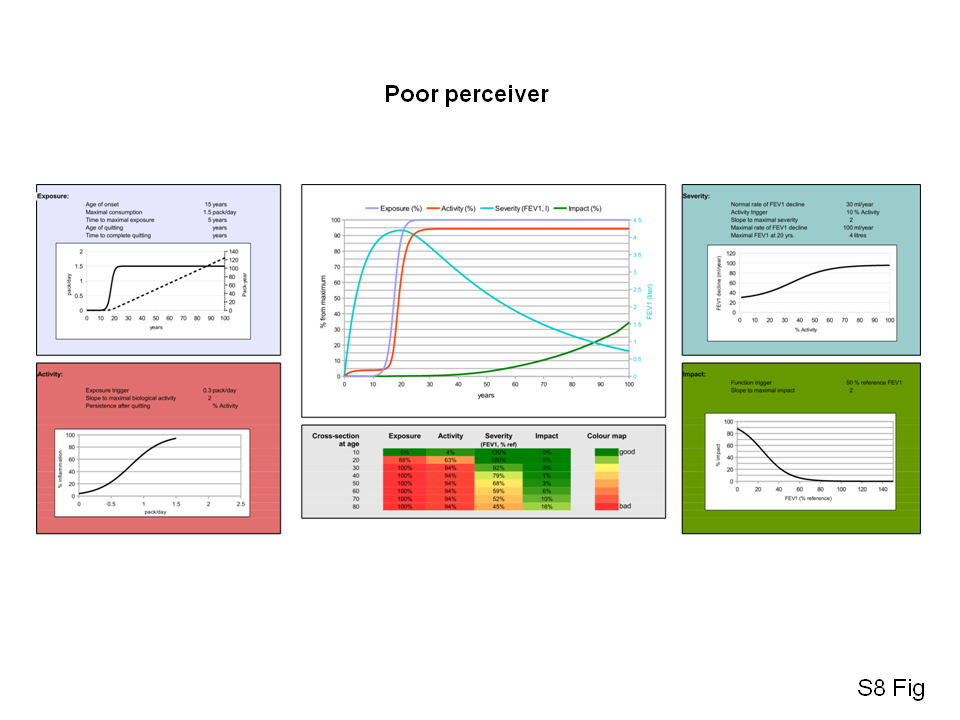

Supplement: S8 Fig — For further explanations, see text. (TIF) [file pone.0185502.s008.tif]
