# Supplementary material for: The EASI model: A first integrative computational approximation to the natural history of COPD
Source: PLoS One. 2017 Oct 10;12(10):e0185502. doi: 10.1371/journal.pone.0185502 (PMC5634586; doi:10.1371/journal.pone.0185502)
Supplement: S2 File — (DOC) [file pone.0185502.s010.doc]

August 29, 2026

**The EASI model: a first integrative computational approximation to the natural history of COPD**

Alvar Agustí¶*^1-3^, Albert Compte¶^2^, Rosa Faner^2,3^, Judith Garcia-Aymerich^4-7^,

Guillaume Noell^2,3^, Borja G. Cosio^3,8^, Robert Rodriguez-Roisin^1-3^,

Bartolomé Celli^9^, Josep Maria Anto^4-7^

¶ These authors contributed equally to this work

**Word count**: 1,3970 words; **References**: 13; **Tables**: 0; **Figures** 8; **Spreadsheet**: 1.

**METHODS**

***E*xposure module**

Briefly, this module computes smoking exposure *E* (t) depending on 5 parameters (Table 1): age of smoking onset, daily tobacco consumption (pack/day), time (yrs.) to maximal exposure, age of smoking cessation (if ever) and the time to complete quitting (if ever). EASI calculates and displays the life-time cumulative smoking exposure (pack-years) (Fig 1, upper left).

*E*(*t*) is defined as the product of two sigmoid (logistic) functions of time, given by:

$$E(t)=E_{\max}\frac{1}{1+e^{-\frac{t-t_{1}}{\sigma_{1}}}}\frac{1}{1+e^{\frac{t-t_{2}}{\sigma_{2}}}}$$

Where *E_max_* is the maximum tobacco consumption per day achieved (pack/day), *t*_1_ is the age of 50% of maximal exposure after starting to smoke, and *t*_2_ is the age of 50% of maximal exposure when quitting. The parameters σ_1_ and σ_2_ define the duration of the transition from not smoking to smoking and *vice versa*, respectively. All these time-related parameters are expressed in years. To facilitate their use and interpretation in our application, we redefine these parameters in the spreadsheet (Fig 1) as: age of onset (= *t*_1_ – 2 σ_1_), time to maximal exposure (= 4 σ_1_), age of quitting (= *t*_2_ – 2 σ_2_), and time to complete quitting (= 4 σ_2_).

***A*ctivity module**

The term “*A*ctivity” refers here to the complex inflammatory response elicited by *E* [1, 2]. In EASI, we simplified it by using a biological activation measure (*A*) that lumps up the effects of many different inflammatory events, this is, without explicitly detailing specific patho-biologic events [3, 4]. Specifically, in EASI *A* is determined by the amount of *E* (packs/year) required to trigger it, as well as by the maximal slope of *A* increase by E. EASI also considers the possibility that *A* may persist after quitting smoking [4]  (Fig 1,lower left).

To model *A*t, we used first-order linear ordinary differential equation as follows:

$$\tau_{A}\frac{dA}{dt}=-A+\left(1-A_{\infty}\right)A_{ss}\left(E\right)+A_{\infty},\text{ with }A_{ss}\left(E\right)=\frac{1}{1+\text{exp}\left[\frac{-\left(E-E_{1}\right)}{\sigma_{e}}\right]}$$

Where *E*_1_ is the value of *E*xposure (pack/day) at which *A* reaches 50%, σ*_e_* (pack/day) is the range of *E*xposure over which Activity (*A*) increases by a factor *e*, τ*_A_* = 2 years is the time scale of *A* dynamics, and $A_{\infty}$ is the persistence of inflammatory activity after quitting (expressed as a fraction, i.e. $A_{\infty}<1$). By definition $A_{\infty}$is 0 before quitting at *t*_2_. These mathematical parameters were defined in the spreadsheet as: Activity trigger (= *E*_1_ – 2 σ*_e_*), slope to maximal Activity (= 0.25 *E_max_*/σ*_e_*, this is the maximal slope of *A_ss_*(*E*), at *E* = *E*_1_) and persistence after quitting (=100 $A_{\infty}$). *A_ss_*(*E*) is represented in the graph in the Activity module (bottom left panel in Fig 1). For clarity, the fractions expressed by *A*(*t*), $A_{\infty}$ and *A_ss_*(*E*) are entered in the spreadsheet in percent value (i.e. multiplied by a factor 100).

***S*everity module**

*Severity* is defined in EASI by the FEV_1_ value at a given age [2]. It depends on the rate of FEV_1_ decline, which is determined by *A(t)*, as well as on the maximum FEV_1_ value achieved in early adulthood [5] (Fig 1). Accordingly, modifiable *S* input parameters include (Table 1) the maximal FEV_1_ attained at 20 years of age, the normal rate of FEV_1_ decline, the level of *A* that initiates abnormal FEV_1_ decline, the steepness of such relationship and the rate of FEV_1_ decline achieved at maximal A (Fig 1, upper right).

Accordingly, *S*(*t*), which represents the extent of lung function (FEV1) available at a given time point [2] which, in turn, is determined by the rate of FEV1 decline through life [6] and by the maximum FEV1 value achieved in early adulthood [5].

**Rate of FEV1 decline**

The rate of FEV1 decline *r* is directly imposed by *A*(t) (given by $r=S_{\max} \tau_{s}^{-1}(A)$and plotted graphically in the Severity module of the application) *via* the following first-order linear ordinary differential equation:

$\tau_{s}(A)\frac{dS}{dt}=-S+Ke^{-t/\tau_{x}}$, with $\tau_{s}^{-1}(A)=S_{\max}^{-1}r_{0}+\left(\frac{1}{1+e^{-(A-A_{1})/\sigma_{a}}}-\frac{1}{1+e^{A_{1}/\sigma_{a}}}\right)S_{\max}^{-1}\left(r_{\max}-r_{0}\right)$

where the parameters *r*_0_ and *r_max_* (ml/year) represent the rate of FEV1 decline in healthy individuals (i.e. for *A*=0) and in individuals with maximal inflammatory response (i.e., for *A* → ), respectively, and *S*_max_ is the maximal lung volume at age 20 (see below). As for other logistic functions, *A*_1_ defines de point of *A* for which FEV1 decline is at 50% of its possible range, and σ*_a_* is the range of *A* values around *A*_1_ over which FEV1 decline ($\tau_{s}^{-1}(A)$) changes by approximately one third of its total allowed range *r_max_* - *r*_0_.

In our application, these parameters are defined as: normal rate of FEV1 decline (*r*_0_ ml/year), severity trigger (= *A*_1_ – 2 σ*_a_*), slope to maximal severity (= 25/σ*_a_*, this is the maximal slope of $\tau_{s}^{-1}(A)$, at *A* = *A*_1_), and maximal rate of FEV1 decline (*r_max_*, in ml/year).

**Peak FEV1 value achieved in early adulthood**

The level of lung function (FEV1) achieved in early adulthood is a key determinant of lung function later in life [5]. To consider this factor in the EASI model, we included two additional parameters: τ*_x_*, which controls FEV1 increase after birth, and *K*, which controls the maximum function achieved. Given *r*_0_ (see above), we fixed τ*_x_* so that FEV1 function *S*(*t*) had its maximum at 20 years of age for healthy non-smokers (*A* = 0). This was achieved by choosing τ*_x_* (in years) satisfying$20=\tau_{x}\left(S_{\max}^{-1}r_{0}\tau_{x}-1\right)^{-1}\ln\left(S_{\max}^{-1}r_{0}\tau_{x}\right)$. On the other hand, *K* was selected so that the maximal lung function achieved at age 20 by healthy non-smokers was *S_max_*. This led to:

$$K=S_{\max}\frac{\left(S_{\max}^{-1}r_{0}\tau_{x}\right)^{-1}-1}{e^{-20S_{\max}^{-1}r_{0}}-e^{-20/\tau_{x}}}$$

In our application, *S_max_* is given by the parameter entry “maximal FEV1 at 20 yrs of age”, in liters.

***I*mpact module**

The term *I*mpact refers here to the level of dyspnea perceived by the patient as a result of *S* [2]. To model it, *S(t)* feeds the *I*mpact module, which computes *I(t)* as the relaxation towards a sigmoid function of *S(t)* (Fig 1, lower right). EASI allows the independent set up of the threshold of *S* to start generating *I,* and the slope to maximal *I* (Table 1). Specifically, the *I*mpact module plots *I*(*t*) as a function of *S*(*t*) through the following first-order linear ordinary differential equation:

$\tau_{i}\frac{dI}{dt}=-I+I_{ss}\left(f_{S}\right)$ , with $f_{S}=\frac{S(t)}{S_{ref}(t)}$ , and $I_{ss}(f_{S})=\frac{100}{1+e^{\frac{f_{S}-f_{1}}{\sigma_{s}}}}$

where *S_ref_*(*t*) is the reference FEV1 value at age *t* [5], *f_1_* is the fraction of the reference FEV1 value at which the perceived *I*mpact reaches the value 50%, σ*_s_* is the range of *f_S_* over which the perceived *I*mpact increases by a factor *e*, and τ*_i_* is the time scale of the *I*(*t*) dynamics, which we set to τ*_i_* = 1 year. Because the data provided in the literature for *S_ref_* does not include data for newborns [7], we interpolated between 0.2 and 2.8 liters of FEV1 in the interval between 0 and 6 years of age.

In our application, these parameters are defined in the *I*mpact module as: impact trigger (=100 (*f*_1_ – 2 σ*_s_*), as % reference FEV1), and slope to maximal impact (= 0.25/σ*_s_*, this is the maximal slope of *I_ss_*(*f_S_*), at *f_S_* = *f*_1_). *I_ss_*(*f_S_*) is represented in the graph in module Impact (right bottom panel in Fig 1).

**Trajectories (natural histories) of COPD**

EASI displays the life-time trajectory of *E(t), A(t), S(t) and I(t)*) (Fig 1, upper central), as well as a cross-sectional (by decade) heat-map (Fig 1, lower central) calculated for each different scenario.

***In silico* calibration of EASI**

To implement the EASI computational model and run a large number of simulations (in order to model population data) we used a Matlab custom code (The Mathworks, Inc.) to: *(1)* determine clinically reasonable ranges for the 15 parameters used (Table 1) by generating 1,000 random models (S1Fig) that had a mean FEV_1_ and coefficient of variation values (by age) that matched those predicted in the non-smoking general population for a 1.75 meter-high male [7] (S2 Fig); *(2)* compare the distribution of FEV_1_ values at the age of 60 years from 1,000 non-smoking models with that expected in a healthy 1.75 m height, never-smoker male (mean FEV_1_ value, 3.2 Litres with a lower limit of normal (LLN) of 2.4 Liters) [7]. We observed that 92% of these models predicted FEV_1_ values above this LLN (S3 Fig); *(3)* constrain the mean FEV_1_ decline and the FEV1 coefficient of variation predicted by these models when persistently exposed to tobacco (from age 12-18 years on) to be within the range of previously reported observations (S2 Fig) [8-11]; and, finally, *(4)* use these 1,000 continuous smokers’ models to estimate the distribution of predicted FEV_1_ values at the age of 60 years below the LLN. We sought that 50% of them were below the LLN (S3 Fig), an estimate that is also in line with previous observations in the general population [8].

**REFERENCES**

1. Hogg JC, Chu F, Utokaparch S, Woods R, Elliott WM, Buzatu L, et al. The Nature of Small-Airway Obstruction in Chronic Obstructive Pulmonary Disease. The New England Journal of Medicine. 2004;350(26):2645-53.

2. Agusti A, Celli B. Avoiding confusion in COPD: from risk factors to phenotypes to measures of disease characterisation. Eur Respir J. 2011;38(4):749-51. doi: 38/4/749 [pii];10.1183/09031936.00062211 [doi].

3. Hautamaki RD, Kobayashi DK, Senior RM, Shapiro SD. Requirement for macrophage elastase for cigarette smoke-induced emphysema in mice. Science. 1997;277(5334):2002-4.

4. Cosio M, Saetta M, Agusti A. Immunological aspects of COPD. New England Journal Medicine. 2009;360:2445-54.

5. Lange P, Celli B, Agusti A, Boje Jensen G, Divo M, Faner R, et al. Lung-Function Trajectories Leading to Chronic Obstructive Pulmonary Disease. New England Journal of Medicine. 2015;373(2):111-22.

6. Fletcher C, Peto R. The natural history of chronic airflow obstruction. Br Med J. 1977;1(6077):1645-8.

7. Quanjer PH, Stanojevic S, Cole TJ, Baur X, Hall GL, Culver BH, et al. Multi-ethnic reference values for spirometry for the 3-95-yr age range: the global lung function 2012 equations. European Respiratory Journal. 2012;40(6):1324-43.

8. Kohansal R, Martinez-Camblor P, Agusti A, Buist AS, Mannino DM, Soriano JB. The Natural History of Chronic Airflow Obstruction Revisited: An Analysis of the Framingham Offspring Cohort. Am J Respir Crit Care Med. 2009;180:3-10. doi: 200901-0047OC [pii];10.1164/rccm.200901-0047OC [doi].

9. Vestbo J, Edwards LD, Scanlon PD, Yates JC, Agusti A, Bakke P, et al. Changes in Forced Expiratory Volume in 1 Second over Time in COPD. New England Journal of Medicine. 2011;365(13):1184-92.

10. Jenkins CR, Jones PW, Calverley PM, Celli B, Anderson JA, Ferguson GT, et al. Efficacy of salmeterol/fluticasone propionate by GOLD stage of chronic obstructive pulmonary disease: analysis from the randomised, placebo-controlled TORCH study. Respir Res. 2009;10(1):59. doi: 1465-9921-10-59 [pii];10.1186/1465-9921-10-59 [doi].

11. Tashkin DP, Celli B, Senn S, Burkhart D, Kesten S, Menjoge S, et al. A 4-Year Trial of Tiotropium in Chronic Obstructive Pulmonary Disease. The New England Journal of Medicine. 2008;359(15):1543-54.

12. Anthonisen NR, Connett JE, Murray RP. Smoking and lung function of Lung Health Study participants after 11 years. Am J Respir Crit Care Med. 2002;166(5):675-9.

13. Scioscia G, Blanco I, Arismendi E, Burgos F, Gistau C, Foschino Barbaro MP, et al. Different dyspnoea perception in COPD patients with frequent and infrequent exacerbations. Thorax. 2017;72(2):117-21. doi: 10.1136/thoraxjnl-2016-208332.

**ON-LINE FIGURE LEGENDS**

**S1 Fig. Distribution of values taken by each of the 15 model parameters in the *in silico* simulation of 1,000 random models in a smoker population** [8]. For non-smoker simulations, parameters in the first row were set to 0 for all 1,000 random models. For further explanations, see text.

**S2 Fig**. **A heterogeneous population of 1,000 models** (parameters as *per* S1 Fig) mimics the mean value (panel A) and variability (panel B) of FEV1 observed experimentally in non-smoker males at different ages [7]. FEV1 decay in persistent smoker model simulations (mean decay across 1,000 models: 50 ml/yr.) is also consistent with experimental data [8]. For further explanations, see text.

**S3 Fig**. **Distribution of FEV1 at age 60 years in the random 1,000 models** (parameters as shown in S1 Fig) for non-smoker (grey columns) or smoker (white columns) models. Solid vertical line marks the reference FEV1 value reported in the literature for a male of height 1.75 m, whereas dotted vertical line marks its lower limit of normality [7]. For further explanations, see text.

**S4 Fig. EASI relationships in a susceptible quitter at 45 years of age** [12]. For further explanations, see text.

**S5 Fig. Same as S4 Fig, but the individual now quits smoking at 65 years of age** [12]. For further explanations, see text.

**S6 Fig**. **EASI relationships in a susceptible quitter (at 45 years of age) in whom inflammation (i.e. disease activity) persists after quitting** [4]. For further explanations, see text.

**S7 Fig. EASI relationships in a continuous smoker who had abnormal lung development early in life** [5]. For further explanations, see text.

**S8 Fig. EASI relationships in a susceptible continuous smoker with poor perception of disease impact** [13]. For further explanations, see text.
